# Role of the HSP70 Co-Chaperone SIL1 in Health and Disease

**DOI:** 10.3390/ijms22041564

**Published:** 2021-02-04

**Authors:** Viraj P. Ichhaporia, Linda M. Hendershot

**Affiliations:** Department of Tumor Cell Biology, St. Jude Children’s Research Hospital, Memphis, TN 38105, USA; viraj.ichhaporia@emdserono.com

**Keywords:** endoplasmic reticulum, HSP70 chaperones, SIL1, BiP/GRP78/HSPA5, unfolded protein response, Marinesco-Sjögren syndrome, neurodegeneration, skeletal muscles, metabolism, glioma, chemical chaperones, gene therapy

## Abstract

Cell surface and secreted proteins provide essential functions for multicellular life. They enter the endoplasmic reticulum (ER) lumen co-translationally, where they mature and fold into their complex three-dimensional structures. The ER is populated with a host of molecular chaperones, associated co-factors, and enzymes that assist and stabilize folded states. Together, they ensure that nascent proteins mature properly or, if this process fails, target them for degradation. BiP, the ER HSP70 chaperone, interacts with unfolded client proteins in a nucleotide-dependent manner, which is tightly regulated by eight DnaJ-type proteins and two nucleotide exchange factors (NEFs), SIL1 and GRP170. Loss of SIL1′s function is the leading cause of Marinesco-Sjögren syndrome (MSS), an autosomal recessive, multisystem disorder. The development of animal models has provided insights into SIL1′s functions and MSS-associated pathologies. This review provides an in-depth update on the current understanding of the molecular mechanisms underlying SIL1′s NEF activity and its role in maintaining ER homeostasis and normal physiology. A precise understanding of the underlying molecular mechanisms associated with the loss of SIL1 may allow for the development of new pharmacological approaches to treat MSS.

## 1. Introduction

**Figure 1:**
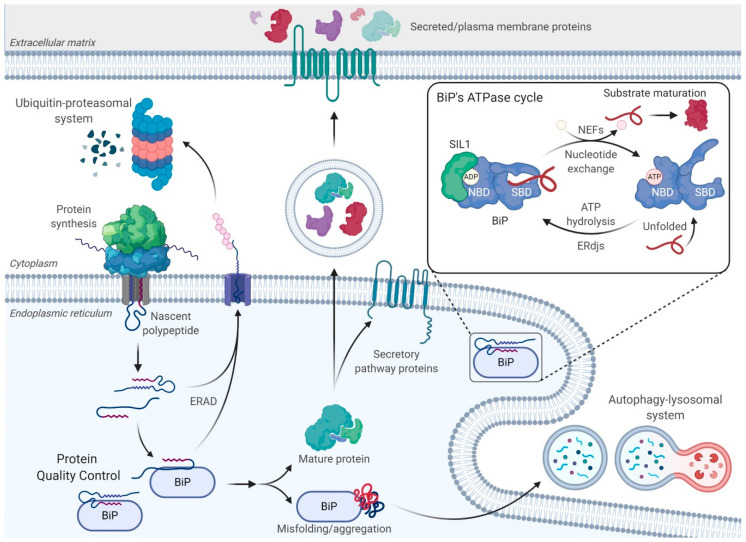
Maintaining endoplasmic reticulum (ER) homeostasis and the ATPase cycle of BiP. Nascent secretory pathway proteins enter the ER in an unfolded state and immediately encounter the ER-resident molecular chaperones, such as the heat shock protein chaperones, BiP, GRP94, and GRP170, as well as the lectin chaperones, Calnexin and Calreticulin. Both of these chaperone families and their associated machineries together ensure that the nascent proteins fold correctly, upon which they can continue their journey along the secretory pathway to their final destination. However, proteins can fail to mature properly. These aberrant proteins are recognized by the same molecular chaperones and retrotranslocated to the cytosol for degradation by the 26S proteasome in a process called ER-associated degradation (ERAD). Aberrantly folded proteins can also be cleared by the autophagy–lysosomal system. (Inset) BiP depends on ATP-induced conformational changes to participate in multiple ER functions. BiP’s ATPase cycle is regulated by two families of co-chaperones, ER-localized DnaJ-like proteins (ERdjs) and nucleotide exchange factors (NEFs). ERdjs enable BiP to associate with its client proteins stably. However, proteins must be released from BiP in order to fold, a process mediated by NEFs, such as SIL1 and GRP170. The released clients can then fold, undergo protein maturation, and traverse the secretory pathway. Created with BioRender.com.

Nearly a third of human genomic open reading frames encode proteins that are residents of single-membrane-bound organelles, the plasma membrane, or the extracellular space [1]. The nascent polypeptide enters the endoplasmic reticulum (ER) co-translationally, where it undergoes processing and post-translational modifications, which may include cleavage of the signal sequence, N-linked glycosylation, disulfide bond formation, and assembly of subunits in the case of multimeric proteins [2]. These manipulations occur as the protein begins to fold into its native structure and can guide the success of the protein-folding process. A dedicated ER protein quality control system (ERQC) ensures the fidelity of protein maturation (Figure 1). It is equipped with multiple molecular chaperones and folding factors that serve to shelter the nascent polypeptide chain entering the ER and prevent its misfolding while promoting efficient maturation to the native state. The ER consists of two major families of molecular chaperones with broad client specificities, the heat shock protein (HSP) chaperones [3] and the glycan-binding lectin chaperones [4], both of which are complemented by a diverse set of co-chaperones. These chaperone families are further assisted by ER-resident protein folding enymes, including peptidyl prolyl *cis/trans* isomerases, which promote isomerization of the *cis* and *trans* peptide bond between prolines and the preceeding amino acid [5,6], and more than 20 ER-localized oxidoreductases that catalyze the formation, reduction, and/or isomerizaton of disulfide bonds between cysteines [7]. Through their interaction with these chaperone systems, unfolded and misfolded proteins are also prevented from being transported through the secretory pathway, thus ensuring that only native proteins will reach their final destination [8].

In spite of the vast cellular resources dedicated to protein folding, a fraction of nascent polypeptides fails to attain their native structure. More than 100 protein-folding diseases have been identified, which result from mutations in individual proteins that disrupt their ability to reach a native state in the ER, and much less frequently, mutations in the ERQC apparatus itself [9]. The resulting aberrantly folded proteins must be identified and degraded by the cell through either the ubiquitin–proteasomal system (UPS) or the autophagy–lysosomal machinery (Figure 1) [10]. In the ER, terminally misfolded and unassembled subunits of multimeric complexes are recognized by some of the same ER chaperones and components of the ERQC machinery [11]. The chaperones maintain the solubility of misfolded proteins for transfer through the retrotranslocon, a protein-conducting channel in the ER membrane. As a misfolded protein begins to emerge into the cytosol, it is ubiquitinated by integral membrane ubiquitin ligases, which allows it to be extracted from the ER by the p97 AAA+ ATPase and targeted for degradation by the cytosolic 26S proteasome. This process is referred to as ER-associated degradation (ERAD) [11,12]. Although the retrotranslocon can accommodate misfolded proteins if they remain soluble, protein aggregates forming in the ER cannot be readily cleared by ERAD. Such ERAD-incompetent aggregates can be degraded by the autophagy–lysosomal system [13,14]. Recently, downstream components of the autophagy pathway were shown to play a role in targeting these aggregates to lysosomes in a process referred to as ER-phagy [15,16]. This process involves reticulons, which bend the ER membrane, proteins with LC3-interacting regions (LIR) that interact with LC3, and other conventional autophagic components for delivery to the lysosomes.

## 2. The ER-Resident HSP70 Chaperone, BiP

Two major chaperone families exist in the ER: the HSP70 cognate, BiP, also known as GRP78 or HSPA5 [17,18], and the lectin chaperones, calnexin (CNX) and calreticulin (CRT), which interact with proteins that are modified by N-linked glycosylation [4]. Although BiP acts as a chaperone for non-glycosylated proteins, it can also chaperone glycoproteins in which the glycans are not in proximity to the unfolded domain. The latter group includes unassembled immunoglobulin (Ig) heavy chains, which were the first BiP clients to be identified [18], coagulation factor VIII [19,20], and a number of viral surface proteins [21], to name a few. BiP was the first molecular chaperone from any organelle or species for which its peptide binding specificity was determined. These studies revealed that BiP bound short peptide stretches of 7–9 amino acids in length enriched in hydrophobic amino acids (Hy(W/X)HyXHyXHy) [22,23]. This type of sequence is predicted to occur approximately every 20 amino acids in the average protein, explaining how BiP is able to interact with so many sequence-unrelated proteins, and would be buried upon client folding, revealing why BiP interacted with unfolded proteins but not with those that had reached their native state.

In addition to BiP’s ability to assist in the *de novo* folding of nascent proteins and target misfolded proteins for degradation, it also plays essential roles in most ER functions. For instance, BiP maintains the ER membrane’s permeability barrier by sealing the luminal face of the translocon channels through which secretory pathway proteins enter the ER [24,25]. BiP also regulates the upstream transducers of the unfolded protein response (UPR) by associating with their luminal domains and suppressing their activation in the absence of ER stress [26]. Finally, BiP is a *bona fide* ER calcium-binding protein, and it contributes to approximately 25% of the ER’s calcium stores that are critical for many signaling pathways [27]. Except for BiP’s contribution to ER’s calcium stores, all other functions rely on the ability of BiP to bind and hydrolyze adenosine triphosphate (ATP).

### 2.1. BiP’s ATPase Cycle

Like all HSP70s, the N-terminus of BiP encodes a highly conserved nucleotide-binding domain (NBD) and a substrate-binding domain (SBD) with an alpha-helical lid at the C-terminus. ATP binding results in a conformational change in the HSP70 chaperone in which the SBD docks onto the NBD and opens the SBD’s lid. This conformer has a high on/off rate for client proteins. ATP hydrolysis leads to undocking of the two domains, resulting in the lid closing over the client and producing a low on/off rate for client binding. In this state, the bound client remains unfolded but protected [28]. The removal of ADP from the NBD and rebinding of ATP are required to reopen the lid, allowing the client to be released so it can fold (Figure 1).

Consequently, BiP mutants that either cannot bind ATP or do not undergo the appropriate conformational change upon ATP binding interact stably with nascent proteins and block their folding, assembly, and secretion [29]. In keeping with BiP’s critical contribution to multiple ER functions, it is one of the very few chaperones that is an essential protein. Biallelic deletion of BiP in mice leads to embryonic lethality at day E3.5 due to a peri-implantation defect [26]. Furthermore, BiP is a target of the AB5 subtilase cytotoxin, which is produced by a pathogenic strain of *Escherichia coli* [30]. This toxin enters cells and cleaves BiP in the linker between the NBD and SBD, thus disrupting the allosteric interaction between these domains that control the SBD’s lid opening and closing. Cells infected with this toxin undergo rapid apoptosis [30].

### 2.2. ERdj Co-Factors for BiP

Two families of co-chaperones tightly regulate BiP’s ATPase activity: ER-resident DnaJ-like proteins (ERdjs) and nucleotide exchange factors (NEFs) (Figure 1). DnaJ family members possess the requisite, highly-conserved J domain, which binds directly to a highly conserved pocket on the NBD through the highly conserved HPD motif of the J domain. This leads to an allosteric coupling of the NBD and SBD through their interaction with the linker that joins them, resulting in ATP hydrolysis [31,32]. To date, eight ERdj proteins have been identified in the ER, at least four of which (ERdj3, ERdj4, ERdj5, and ERdj6) can also directly bind to various sequence-unrelated clients [3]. The association of BiP with an ERdj bound to a substrate results in the transfer of the subtrate to BiP and the hydrolysis of the bound ATP molecule to ADP. The low affinity of DnaJ-type proteins for the ADP-bound form of BiP causes the release of the ERdj protein from the BiP–substrate–ERdj complex. It appears that the ability of ERdj proteins to bind specific proteins and transfer them to BiP provides a mechanism for BiP’s involvement in distinct ER functions.

### 2.3. BiP’s Nucleotide Exchange Factors

Importantly, the substrate does not fold while bound to BiP since the SBD’s closed lid restricts the substrate’s conformational freedom. In order to fold, the substrate must be released from BiP, a process that is aided by NEFs. These co-factors introduce a torsional strain in BiP’s NBD, which allows disengagement of the bound ADP molecule by breaking the hydrogen-bonded contacts. ADP release enables the BiP-NBD to engage a new ATP molecule, which causes the SBD to dock onto the NBD and the lid to open, leading to active release of the bound polypeptide substrate. Until recently, all ATP-bound structures for HSP70s were obtained by using ATP hydrolysis mutants, but a clever use of inorganic phosphorus in the crystalization protocol resulted in the identification of an additional ATP-bound structure for wild-type BiP [33]. This structure predicted that ATP binding could “squeeze” a client protein from the SBD before resetting this domain to the high on/off conformer. This mechanism prevents BiP from repeatedly engaging the same substrate and provides a spatial and temporal window in which that substrate may fold [33,34]. There are two ER-resident co-chaperonesthat have been reported to possess NEF activity towards BiP: SIL1 and GRP170.

#### GRP170

GRP170 was originally identified as a stress-inducible protein that associates with BiP and also with several unfolded client proteins in immune lineage cells [35]. It was shown to suppress the aggregation of denatured luciferase, making it a *bona fide* ER chaperone. It possesses regions that are structurally similar to BiP, including a nucleotide-binding domain and an apparent client-binding domain, leading to its designation as a large-HSP70 or HSP110 family member [36]. Subsequently, the ATP-binding property of GRP170/Lhs1p was found to be essential for the translocation of nascent chains into purified microsomes [37] and the yeast ER [38]. The link between its interaction with BiP and the ability to bind ATP led to the discovery that GRP170 and other HSP110 family members also possess NEF activity for BiP [39,40].

## 3. SIL1

### 3.1. Discovery and Expression

In the mid-1990s, several laboratories independently discovered Sil1 in three different organisms. A conditional lethality screen was used in the yeast *Yarrowia lipolytica* to detect cellular components that interacted with the 7S RNA or the signal recognition particle (SRP) during co-translational translocation, which led to the identification of the *Sls1p* gene [41]. Sls1p is an ER-luminal, 54 kDa protein that contains an N-terminal signal sequence and a C-terminal ER-retention motif. Sls1p fractionated with the membranous fraction and was shown to interact with the Sec61p translocation apparatus. Sls1p was induced by ER stresses, such as heat shock or inhibition of glycosylation, and its deletion resulted in decreased maturation of secretory proteins. This protein also bound to the ADP-bound form of Kar2p, the yeast homolog of BiP, and stimulated its interaction with Sec63, a DnaJ family member and a translocon component [42]. Disruption of the interaction between Sls1p and Kar2p significantly affected the secretion of a reporter protein [41,43], leading the investigators to conclude that Sls1p assisted BiP in the translocation of nascent proteins into the ER lumen [41]. Another group working with *Saccharomyces cerevisiae* conducted a screen for genes that would suppress the severe growth defect observed in the *ΔIre1ΔLhs1* yeast double mutants when overexpressed and named this gene the suppressor of *Ire1* and *Lhs1* deletion 1 (Sil1). Sil1p was shown to be a homolog of Sls1p, and a combined deletion of *Sil1p* and *Lhs1* (yeast GRP170) proved to be lethal after causing a total block in protein translocation into the ER [44]. Mammalian (human) SIL1 was discovered in a yeast two-hybrid screen aimed at identifying proteins that interacted with a mutant ATPase domain of BiP and was initially named BiP-associated protein (BAP) [45]. Sequence analysis revealed that BAP is a mammalian homolog of Sls1p and Sil1p and is similar to the cytosolic HSP70-binding protein, HSPBP1. Biochemical data further demonstrated that BAP also had NEF activity for BiP.

### 3.2. Structure, Mechanism of Nucleotide Exchange, and Expression

**Figure 2:**
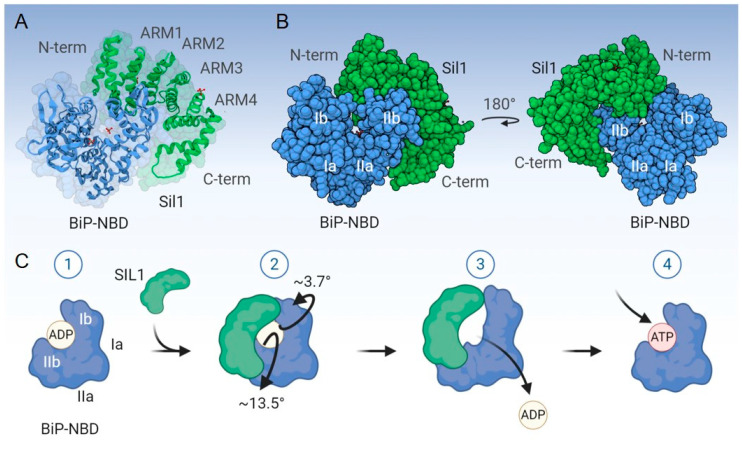
Mechanism of nucleotide exchange by SIL1. (**A**,**B**) Displays the structure of a portion of *S. cerevisiae* Sil1 (Sil1p; green) complexed with the nucleotide-binding domain (NBD) of *S. cerevisiae* BiP (Kar2p; blue); PDB: 3QML. The N-terminus (N-term), C-terminus (C-term), and the four ARM domains of Sil1p are indicated, as are lobes Ia, Ib, IIa, and IIb of BiP’s NBD. (**C**) Demonstrates the mechanism of ADP to ATP exchange mediated by Sil1p. (1) Sil1p associates preferentially with the ADP-bound form of BiP. (2) Sil1p binds lobe IIb (major interaction site) as a “clamp” and also makes contact with lobe Ib (minor interaction site). The interaction of Sil1p with lobe Ib of BiP-NBD serves as the pivot point for Sil1p by which it introduces torsional strain in BiP’s NBD. (3) This, in turn, generates a conformational change in BiP’s NBD, swinging lobes IIb and Ib away from the nucleotide-binding pocket by ~13.5° and ~3.7°, respectively. These conformational changes abolish the hydrogen bonds between ADP and the respective residues from BiP’s NBD, which subsequently releases ADP from BiP and (4) enables BiP to enter a new cycle upon engaging its client substrate. Created with BioRender.com.

Structural data for yeast Sil1p revealed an elongated, “kidney bean”-like molecular shape that consists of 16 α-helices (A1–A16) and lacks β-sheets (Figure 2). The central helices A3-A14 form the armadillo (ARM)-like repeats (ARM1-ARM4) [34], which are named after those found in β-catenin. Each ARM repeat is composed of three α-helices that pack into a superhelix. A crystal structure of yeast Sil1p complexed with the ADP-bound NBD of Kar2p/BiP revealed that the ARM domain of Sil1p wraps around lobe IIb of BiP’s NBD and makes additional contacts with lobe Ib. This interaction causes lobes Ib and IIb to rotate away from each other, leading to ADP release (Figure 2) [34]. Point mutations in the Sil1p-interacting site of Kar2p’s NBD specifically disrupted Sil1p binding but retained Kar2p’s ability to interact with Lhs1/GRP170, the other ER NEF [46], providing the first indication that their mechanisms of NEF activity were different. Homology mapping and comparisons with the structure of cytosolic HSPBP1 indicated that the region of SIL1 encoded by exons 6 and 9 constitutes major BiP-binding sites, and exon 10 encodes a minor interaction site [47].

**Figure 3:**
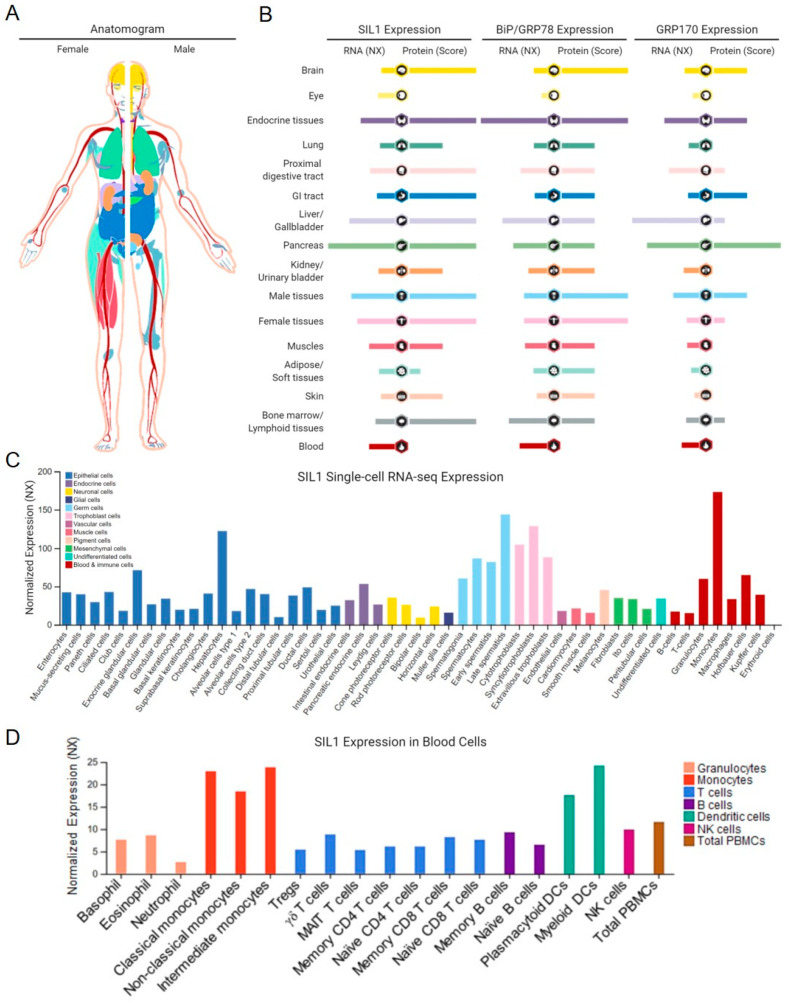
Tissue-specific and cellular expression of SIL1. (**A**) An anatomogram with color-coded tissues used to denote SIL1 expression. (**B**) An overview of normalized RNA expression (NX; RNA-seq data derived from Human Protein Atlas, FANTOM5, and GTEx consortia) and protein scores of SIL1, BiP, and GRP170, respectively, from 16 healthy organ and tissue systems, representing similarities between SIL1 and BiP expression patterns and some differences between those of SIL1 and GRP170. (**C**) Summary of single-cell RNA-seq normalized expression (NX) from 51 cell types. Color-coding varies from (**A**,**B**) and is based on cell types with common functional features. (**D**) SIL1 mRNA expression in 18 blood cell types and total peripheral blood mononuclear cells. Color-coding is based on blood cell type lineages, including B-cells, T-cells, NK-cells, monocytes, granulocytes, and dendritic cells, as well as total PBMC. Data credits: Human Protein Atlas. Data images were obtained from proteinatlas.org/ENSG00000120725-SIL1.

Although SIL1 is ubiquitously expressed, levels vary widely by tissue (Figure 3) [45,48,49,50,51,52]. The Human Protein Atlas (www.proteinatlas.org) shows that the expression pattern of SIL1 mirrors that of BiP even more closely than that of GRP170, which is a known UPR target, as is BiP [53,54]. Perhaps the different expression pattern for GRP170 is due to its dual function as a chaperone and a NEF for BiP. *SIL1* expression at a single-cell level has also been determined in a number of tissues, which does not strictly correlate with the secretory capacity of the cell, and tissues displaying the highest relative levels of SIL1 are not necessarily those most affected by loss of its function [47,55]. This might imply an additional function, which has been demonstrated for Sil1p [56], or an as yet undiscovered role of SIL1 in certain tissues. Understanding this reason for variation in tissue expression will require further studies.

## 4. Marinesco-Sjögren Syndrome

**Figure 4:**
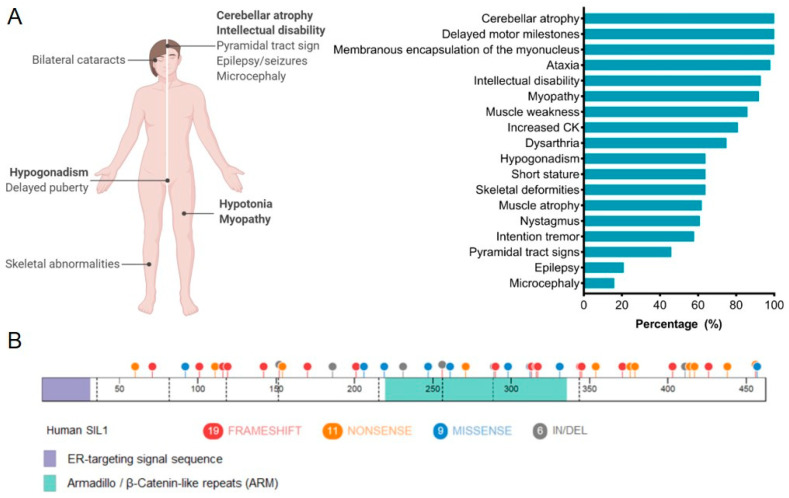
The genetic and clinical heterogeneity associated with Marinesco-Sjögren syndrome (MSS). (**A**) The spectrum of clinical features associated with MSS and their relative frequencies. Adapted from Krieger et al. [60]. (**B**) The SIL1 protein cartoon demonstrates 45 MSS-associated mutations (1 intronic mutation is not displayed). Mutations are indicated as frameshift (red), nonsense (orange), missense (blue), and in/del (grey). Lilac, ER-targeting signal sequence; light green, armadillo-like repeats. Created with BioRender.com.

In 1931, Marinesco and colleagues first described a syndrome characterized by cerebellar ataxia, congenital cataracts, and physical as well as mental retardation [57]. Nearly 20 years later, Sjögren expanded the patient population and reported this disorder to be transmitted in an autosomal recessive manner [58]. Subsequent reports on individuals with this group of symptoms, which became known as Marinesco-Sjögren syndrome (MSS; OMIM: 248800), revealed that this disease occurred equally in males and females and was identified in individuals of numerous nationalities and ethnicities. Today, MSS is defined as a multisystem disorder, but the cardinal features used for a clinical diagnosis include cerebellar dysfunction and ataxia, bilateral cataracts, mental deterioration, impaired physical development, skeletal deformities, and progressive myopathy [59]. Figure 4A displays the relative frequency of symptoms observed in MSS [60]. Cerebellar symptoms represent the most frequent and one of the earliest clinical presentations of MSS, occurring as early as 14 months [61], and are characterized by severe ataxia due to cerebellar degeneration, consisting primarily of Purkinje and granule cell loss [55]. Bilateral cataracts are nearly as frequent as the occurrence of cerebellar ataxia and precipitate during infancy with an associated visual impairment [59], although the molecular etiology of cataract formation in the case of MSS has remained elusive. Mental deficiencies in MSS present as learning disabilities and regressive tendencies due to chronic atrophy of nerve cells rather than an inflammatory process, as first reported by Marinesco [57,59]. Progressive atrophy of skeletal muscles and the accompanying hypotonia are also frequent symptoms of MSS [62]. Ultrastructural analyses of muscle tissue demonstrated variations in myofiber size and morphology, degeneration and regeneration of fibers, as indicated by internalization of myonuclei, and abnormal membrane structures surrounding the nucleus, which came to be recognized as a hallmark of the MSS-associated myopathy [63]. Evidence of an ongoing autophagic phenomenon was also identified, hinting at possible metabolic defects in muscles from individuals with MSS [64,65]. The atrophy of type I skeletal muscles is a distinct feature of this disease, which spares the heart and type II fibers [59]. The myopathy observed in MSS patients was initially proposed to lack a neurogenic component that is frequently found in other myopathies [66,67], but more recent data suggest otherwise [68].

Nearly 70 years after the first report of MSS, the gene responsible for the majority of cases was mapped to human chromosome 5q31 [69], and soon after, two groups independently identified mutations in the *SIL1* gene within the 5q31 locus [47,55]. Currently, 46 MSS-associated mutations have been described in *SIL1*, including premature truncations, frame-shift mutations, in-frame deletions, and missense mutations (Figure 4B) [55]. Many of these mutations are expected to result in the deletion of a majority of the SIL1 protein or at least those portions of SIL1 that are predicted to interact with BiP [47]. When exogenously expressed, multiple SIL1 mutants were found to be either aggregation-prone or rapidly turned over by ERAD [60,70,71,72,73,74,75]. However, approximately 40% of individuals diagnosed with classical MSS pathologies do not have mutations in *SIL1*. Since these patients display nearly identical clinical features, investigators have checked for mutations in other genes that function in the same pathway as SIL1. An extended genetic analysis conducted on 18 MSS-patient samples with normal SIL1 expression discovered no evidence of mutations, polymorphisms, or altered expression of either *HYOU1*/*GRP170* or *HSPA5*/*BiP* [70]. The gene encoding the alanyl tRNA synthetase (*AARS*), which causes an MSS-like phenotype in mice, was also examined and not found to be associated with MSS [70]. To date, the affected gene(s) responsible for the remaining 40% of MSS cases remains unknown.

### 4.1. Mechanistic Insights into MSS-Associated Pathologies

Soon after *SIL1* was identified as the gene responsible for ~60% of MSS cases, two mouse models of *Sil1* disruption were developed. In both models, intron 7 of the murine *Sil1* gene was disrupted. This occurred either by the insertion of an ETn retrotransposon, which resulted in an in-frame stop 96 nucleotides after the retrotransposon coding sequence, or by integration of an in-frame β-geo gene-trap cassette that caused truncation of the wild-type *Sil1* transcripts after exon 7 and subsequent fusion with the β-geo part of the construct, resulting in a significant extension of this protein [76].

Although there were differences in the resulting SIL1 chimeric proteins and each genetic manipulation was produced in a distinct murine genetic background (CxB5/ByJ versus C57BL/6J, respectively), both mice recapitulated the majority of the cardinal features associated with MSS [76]. These mice have provided valuable tools for obtaining molecular insights into MSS-associated pathologies and the role of SIL1 in maintaining ER homeostasis.

#### 4.1.1. The Neurological Features of MSS and the Role of SIL1

The first instance where SIL1-deficient mice provided mechanistic insights into MSS-related pathologies came from studies to understand the cerebral deterioration and severe ataxia that are hallmarks of the disease [77]. Both mouse models developed ataxia with 100% penetrance. In fact, the mouse with the retrotransposon insertion into the *Sil1* gene was referred to as the “woozy” mouse (*Sil1^wz^*) due to the early onset of ataxia [76]. The mice also displayed Purkinje cell loss, characterized by the accumulation of membranous autophagosomes, ubiquitinated protein aggregates, activation of the UPR, and ultimately apoptosis, demonstrating that MSS represented a protein folding disorder. SIL1 was found to be widely expressed in the murine brain. The cerebellar Purkinje cells in lobules I-IX seemed to have similar levels of SIL1 expression in the wild-type mice, but the loss of SIL1 severely affected only lobes I-VIII of the neocerebellum while sparing lobule X and caudal lobule IX of the vestibulocerebellum [76].

Although BiP deficiency is embryonic lethal, the non-lethal phenotype of SIL1-deficient mice was hypothesized to be due to the redundancy of SIL1 with GRP170, which also demonstrates nucleotide exchange activity towards BiP [78,79,80,81]. Consistent with this possibility, the reduction of GRP170 expression worsens the cerebellar phenotype in *Sil1*-disrupted mice and adversely impacts the previously unaffected IX and X lobules. Similarly, transgenic overexpression of GRP170 in Purkinje cells completely rescued their degeneration, indicating that GRP170 is likely a modifier of this disease [82]. Although endogenous GRP170 is upregulated in the Purkinje cells of SIL1-deficient mice, this increase did not match the levels of transgenic overexpression, which may explain the inability of endogenous GRP170 to compensate for SIL1 loss beyond a certain threshold. The affected tissue systems in humans and mice might not be able to upregulate GRP170 sufficiently to prevent pathologies, because unresolved ER stress often leads to the activation of apoptosis [83]. Reducing levels of one of the ER DnaJ family proteins, DNAJC3/ERdj6, in the *Sil1*-disrupted mice alleviated the cerebellar neurodegeneration and attenuated ER stress, suggesting that reducing the amount of BiP in the ADP-bound form is beneficial in the context of SIL1 loss [82].

Mild to moderate intellectual disability is another cardinal feature of MSS that precipitates relatively early in the course of this disease [60]. In situ hybridization has demonstrated that *Sil1* mRNA is co-expressed with BiP in a spatiotemporal manner during mouse brain development [60,73,74]. Ex vivo depletion of SIL1 in ventricular zone progenitor cells isolated from murine embryonic brains caused an abnormal cellular morphology, aberrant neuronal migration pattern with delayed kinetics, and slower axonal growth. This phenotype resembles that of BiP knockdown in the ventricular zone progenitor cells, as well as knock-in expression of a BiP mutant in mice [60,73,74]. Human SIL1 overexpression rescued the migration defects observed upon murine Sil1 depletion, whereas three MSS-associated SIL1 mutants failed to do so, highlighting the pathophysiological significance of these SIL1 mutant proteins in MSS. The perturbation of the cortical neuronal cytoarchitecture, combined with the potential effects of SIL1 loss on neuronal protein quality control mediated by BiP, may cause the varying degrees of intellectual impairment observed in MSS.

#### 4.1.2. The Myopathy in MSS

Multiple studies have characterized the highly conserved myopathic changes in patient biopsies and confirmed muscular dystrophy in MSS with internalized myonuclei, variation in myofiber size, the presence of atrophic and hypertrophic fibers, membranous autophagic whorls, and a membranous structure encapsulating the nucleus. In addition, a depletion of the predominantly glycolytic, type IIB myofibers and mitochondrial abnormalities was uniformly present, suggesting that disturbed lysosomal function contributes to the muscle pathology in MSS [62,63,64,65,84,85]. Myopathy is a distinct feature in MSS since it spares the heart, which is commonly affected in spinocerebellar degenerative disorders [59]. Two studies using different mouse models demonstrated that *Sil1* disruption in mice phenocopies the ultrastructural features of the progressive myopathy observed in MSS patients and have proved useful for molecular and longitudinal studies of the myopathy [86,87]. These studies established that loss of SIL1 led to activation of the UPR coinciding with the onset of muscle weakness, which drove the upregulation of numerous ER chaperones, co-chaperones, and ERAD components, suggesting that the basal levels of these protein folding machineries were sufficient to maintain ER homeostasis before the onset of myopathy [86,87]. At the same point, there was evidence of an autophagic impairment [86,87]. Autophagy plays a key role in regulating skeletal muscle mass, and the absence of an unfettered autophagic response leads to loss of muscle mass and force, protein aggregation, the presence of abnormal membranous structures, and dilated sarcoplasmic reticulum [88], which are all in keeping with the observations in *Sil1*-disrupted mice and MSS-patient-derived biopsies [47,64,65,87].

An unbiased proteomics approach conducted at the physiological onset of decreased muscular strength revealed global perturbations in cellular proteostasis, affecting all major organelles and multiple pathways critical for skeletal muscle function [86,87]. Notably, the maturation of insulin and IGF-1 receptors (IR and IGF1R, respectively), which fold and assemble in the ER, was reduced in both *Sil1^Gt^* quadriceps and in C2C12 murine myoblasts with a CRISPR/Cas9-mediated *Sil1* knockout [72], along with a concomitant increase in the GLUT4 glucose transporter. IR and IGF1R are essential regulators of skeletal muscle protein- and glucose-homeostasis via activation of the downstream PI3K-AKT-mTOR signaling pathway. In the face of decreased IR and IGF1R levels, the PI3K-AKT-mTOR pathway was paradoxically activated in ad libitum fed *Sil1^Gt^* mice under steady-state conditions [86,87]. A similar compensation has been observed in mice with a muscle-specific dual knockout of IR and IGF1R (MIGIRKO), which display basal activation of PI3K-AKT-mTOR signaling and increased surface expression of the GLUT1 and GLUT4 glucose transporters [89].

Acute skeletal muscle atrophy occurs in numerous other conditions, such as cancer, Cushing’s syndrome, denervation, diabetes, disuse atrophy, fasting, sepsis, and uremia, and is driven by the activation of the FOXO transcription factors [90]. These transcription factors upregulate the expression of two notable E3 ubiquitin-ligases, Atrogin-1 and MuRF1, which serve as master regulators of the ubiquitin–proteasomal process [91]. However, there was no evidence of a sustained upregulation of either of these E3 ubiquitin-ligases in *Sil1^Gt^* skeletal muscles, and instead these proteins were modestly decreased. This is consistent with the actual inhibition of FOXO’s transcriptional activity that normally occurs upon activation of PI3K-AKT-mTOR signaling. The myopathy observed in *Sil1^Gt^* mice also mimics numerous features of sarcopenia—the aging-related progressive loss of skeletal muscle mass and function [92]—and AKT-mediated downregulation of Atrogin-1 and MuRF1 is one such feature of this process [93].

It is worth highlighting that the *Sil1^Gt^* and *Sil1^wz^* mice differ in several significant aspects [72,86,87,94]. Although there were minor changes in the onset and magnitude of UPR activation between the two models, more significantly, the myopathy in the *Sil1^wz^* mouse was diagnosed to have a significant neurogenic contribution, with the degeneration of peripheral nerves and neuromuscular junctions. In contrast, there was no substantial evidence of the involvement of neuromuscular junctions in the *Sil1^Gt^* myopathy. The reason for this difference is unclear but could represent varying effects on individual proteins or the presence of genetic modifiers that differ between these models. Further studies are required to understand the significance of this and its relationship, if any, to MSS.

#### 4.1.3. Metabolic Features Associated with SIL1 Depletion

To the best of our knowledge, glucose metabolism abnormalities have been reported in only two studies to date, involving five MSS patients [69,95]. A modest abnormality in cerebral glucose metabolism was observed in one out of the two patients evaluated [95], increased glucose tolerance was identified in another patient, and insulin-dependent diabetes mellitus was present in two patients [69]. While there is a lack of in-depth studies to evaluate possible MSS-associated metabolic defects, data from murine studies with a loss of SIL1 suggest that metabolism may be more widely affected than currently known. In keeping with the observation of insulin-dependent diabetes mellitus in MSS, studies in *Sil1^wz^* mice found that SIL1 was required to maintain normal insulin levels and pancreatic β cell morphology [94,96]. These mice were more vulnerable to streptozotocin-induced type I diabetes and experienced glucose intolerance upon high-fat diet feeding. Insulin is a known BiP client [97], and it is conceivable that the loss of SIL1 directly or indirectly interferes with BiP release and prevents its secretion, although this was not directly measured. In support of this hypothesis, SIL1 knockdown and overexpression studies in a pancreatic cell line resulted in insulin secretion that corresponded in magnitude to SIL1 levels [94]. 

**Figure 5:**
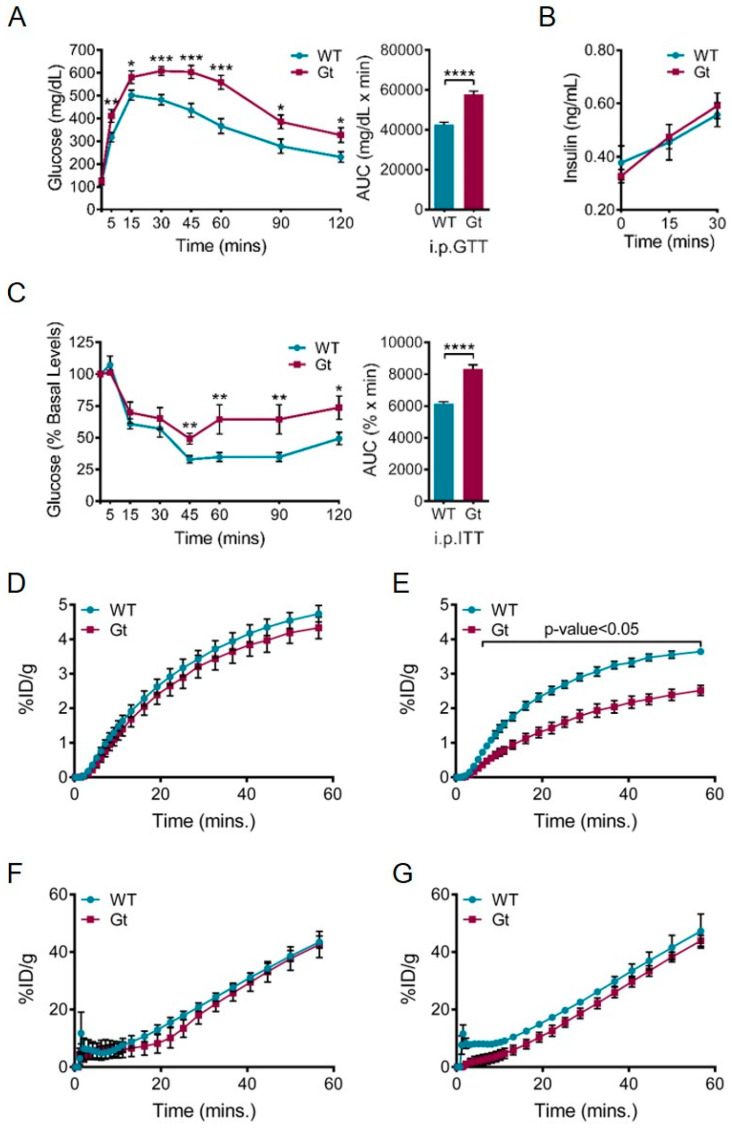
Loss of SIL1 leads to systemic defects in glucose homeostasis in *Sil1^Gt^* mice. (**A**–**C**) *Sil1^Gt^* mice demonstrate glucose intolerance and insulin resistance. Age-matched wild-type and *Sil1^Gt^* mice (*n* = 8–12) were subjected to an (**A**) intraperitoneal glucose tolerance test (i.p.GTT) where d-glucose was administered at a dose of 2 mg/g body weight, or (**C**) an intraperitoneal insulin tolerance test (i.p.ITT) where human insulin was administered at a dose of 0.5 mU/g body weight, following which blood glucose was measured at the indicated time-points. Area under curve (AUC) calculations for each test are displayed next to the respective blood glucose levels. Error bars indicate means ± s.e.m. (**B**) Plasma insulin levels during the initial time-course of the i.p.GTT were measured and plotted. Statistical comparisons were performed using t-tests; *p*-values are indicated as * *p* ≤ 0.05, ** *p* ≤ 0.01, *** *p* ≤ 0.001, **** *p* ≤ 0.0001. (**D**–**G**) Graphical representation of 18F-fluorodeoxyglucose (^18^F-FDG) uptake over 60 min in wild-type (blue) and *Sil1^Gt^* (red) brains (**D**,**E**) and urinary bladders (**F**,**G**) under basal conditions (**D**,**F**) or after an intraperitoneal injection of 0.75 mU/g body weight of human insulin (**E**,**G**; *n* = 4) assayed by PET-µCT. Urinary bladder serves as a control tissue that should not demonstrate differences in ^18^F-FDG uptake between *Sil1^Gt^* and wild-type mice. Statistical differences were computed using unpaired, two-tailed Student’s t-tests and are indicated in the graph. %ID/g, % injected dose per gram body weight.

The defect in *Sil1^wz^* mice hints at a deficiency in insulin production, but not in insulin-mediated glucose uptake because these mice demonstrate normal insulin tolerance. This pattern contradicts the defect observed in *Sil1^Gt^* mice, which display impaired glucose and insulin tolerance (Figure 5A,C), indicating a defect in glucose uptake even when insulin is exogenously supplemented. As a matter of fact, *Sil1^Gt^* mice demonstrated normal insulin production after a glucose challenge (Figure 5B), arguing that these mice do not have a significant problem with folding and secreting insulin but instead have impaired insulin-mediated glucose uptake. In support of this possibility, *Sil1^Gt^* mice display normal glucose uptake under basal conditions in the brain but have impaired insulin-mediated cerebral glucose uptake (Figure 5D–G). It is possible that aberrant glucose uptake in the *Sil1^Gt^* brain may involve reduced IR and IGF1R levels, which are both decreased in *Sil1^Gt^* muscles. Since the *Sil1^wz^* and *Sil1^Gt^* mouse models reproduce many aspects of MSS, it is reasonable to evaluate systemic glucose metabolism in MSS patients more thoroughly in future studies.

#### 4.1.4. Bilateral Cataracts

Bilateral cataracts are nearly as frequent as cerebellar ataxia in individuals with MSS and precipitate during infancy with an associated visual impairment. Numerous reports have detailed the varying characteristics of MSS-associated bilateral cataracts, spanning from fine dot-like depositions to flaky deposits in concentric layers of the cortex and powdery opacities within the nucleus [59]. However, their overall etiology remains elusive. Thus far, neither mouse model has shown evidence of bilateral cataracts, although thorough studies have not been conducted to detect ultrastructural or proteomic changes that might provide insights into this pathology. Of note, *Sil1^Gt^* mice demonstrate unilateral cataracts with infrequent occurrences, suggesting that this phenotype varies between humans and mice lacking SIL1. 

#### 4.1.5. Role of SIL1 in B-lymphocytes

B cells are progenitors to antibody-producing plasma cells, and antibodies represent the best-characterized substrate of BiP. Studies have demonstrated that the expression of BiP mutants that bind but cannot be released from immunoglobulin heavy chains completely inhibits antibody assembly and secretion [98]. Thus, it seemed plausible that antibody production might be affected in individuals with MSS or SIL1-deficient mice. However, antibody responses were unaffected in *Sil1^Gt^* mice that were immunized with keyhole limpet hemocyanin, a highly immunogenic antigen that elicits a T-cell-dependent B-cell immune response, as was ex vivo antibody production in LPS-stimulated splenic lymphocytes [75]. Although SIL1 was significantly upregulated during LPS-mediated differentiation of wild-type lymphocytes, those from *Sil1^Gt^* mice did not compensate by increasing the levels of GRP170 or other chaperones beyond that occurring as part of the differentiation schema. In keeping with this finding, EBV-transformed lymphoblastoid lines (LBLs) from MSS-affected individuals synthesized and secreted comparable amounts of antibody to those from normal controls [75]. In spite of normal antibody production, a second study with LBLs obtained from MSS patients identified a number of ultrastructural anomalies in these cells, including cytoplasmic inclusions and abnormal organellar morphologies [99]. Mass spectrometry analyses on these cell lines revealed alterations in the cytoskeletal, secretory pathway (ER, endosomes, Golgi, lysosomes), mitochondrial, and nuclear proteomes. A subset of these proteins was examined in splenocytes from *Sil1^wz^* mice and found to have similar changes in expression patterns. This is reminiscent of structural abnormalities detected in MSS-patient-derived skin fibroblasts [100], even though this tissue seems to be spared in affected individuals. These findings may indicate a subclinical vulnerability of certain tissues.

### 4.2. Why Does the Loss of SIL1 in Humans and Mice Selectively Affect Some Tissues while Sparing Others?

Clearly, not all tissues are equally affected in either individuals with MSS or the two mouse models in which *Sil1* has been disrupted. Paradoxically, it appears that highly secretory tissues, such as the kidney, liver, placenta, and plasma cells, where one might expect adverse consequences when a component of the chaperone machinery is dysfunctional, are relatively unaffected by SIL1 loss [75]. Instead, the effects are most significant in the Purkinje and glial cells of the cerebellum, the lens epithelial cells, and skeletal muscles [60], which are not generally associated with the synthesis of industrial amounts of secretory proteins that are BiP substrates. The more classical secretory tissues may have a greater number of redundant mechanisms built into their cellular systems to accommodate their large load of ER clients, and therefore, are less affected by cellular stress. This scenario can be exemplified by the differences between motor neurons innervating fast versus slow skeletal muscles [101]. The ER of fast motor neurons express relatively higher levels of ERAD components, such as VIMP, EDEM1, SEL1L, and OS9, and ER stress response components, such as PERK, IRE1, and EIF2α. Conversely, the ER of slow motor neurons express higher levels of chaperones and co-chaperones, such as SIL1, ERP29, CRT, BiP, GRP94, and GRP170 [101]. These fast motor neurons display low excitability but are highly phasic and generate short-lived powerful muscle contractions, whereas the slow motor neurons are smaller in size, highly excitable, and extremely resistant to fatigue. In keeping with the differences in their functional demands, the distinct expression patterns of proteostasis components might elicit dramatically distinct responses upon disruption of ER homeostasis. 

The neocerebellum and the vestibulocerebellum serve as another example of how the molecular makeup of a cellular subtype affects vulnerability upon loss of SIL1. Distinct differences in the expression patterns of multiple proteins in the neocerebellum and vestibulocerebellum of *Sil1*-disrupted mice have been noted [99]. The neocerebellum is highly susceptible to SIL1 deficiency, whereas the vestibulocerebellum is relatively resistant. Proteins with a differential signature between these two cerebellar regions include calmodulin, cytochrome c, α-synuclein, phosphoglycerate dehydrogenase (PHGDH), and ataxin-10 (ATXN10), which have all been associated with neuronal dysfunction to varying degrees during pathological instances. Both PHGDH and ATXN10 were considerably decreased in the neocerebellum of *Sil1^wz^* mice before the onset of cerebellar atrophy, but not in the vestibulocerebellum. Decreases in the expression of these two proteins have been linked to other neurological diseases distinct from MSS [102,103]. While these proteins do not reside in the secretory pathway and are not likely to be BiP clients, it is certainly feasible that differences in their protein levels represent secondary and/or tertiary consequences of SIL1 loss, which in turn contribute to the diverse neurological symptoms that are associated with MSS with varying penetrance.

One intriguing new function described for yeast Sil1, which could affect tissues differentially upon its loss, comes from genetic and biochemical studies showing that yeast Kar2p/BiP is a direct sensor of changes in the ER redox balance. This is mediated via the reversible oxidation of a highly conserved cysteine residue in Kar2p’s NBD (cysteine-63), which enhances its ability to bind to misfolded proteins and prevent protein aggregation by decoupling its holdase activity from regulation by BiP’s nucleotide-bound state [104,105]. In yeast, Sil1p moonlights as a reductase for oxidized Kar2p via two N-terminal cysteines, thereby aiding cells in recovery from oxidative stress [56]. While these cysteines are not present in mammalian SIL1, two other cysteines (cysteines-25 and -29) are present in the signal sequence (..ACFTFCL..), which could serve this function if retained in the mature protein. However, data to demonstrate this have not yet been obtained for mammalian SIL1, which could provide insights into the variations in tissue expression shown in Figure 3. However, perhaps most inconsistent with this possibility is that secretory tissues, which produce high levels of molecular oxygen due to the high rate of disulfide bond formation, are the very tissues that appear to be spared in MSS.

### 4.3. Linkage of SIL1 with Non-MSS Pathologies

Since SIL1 plays a crucial role in maintaining ER homeostasis in neurons, it did not come as a complete surprise when SIL1 was implicated as a modifier in amyotrophic lateral sclerosis (ALS) [101]. SIL1-haploinsufficiency in SOD1 mutant (*SOD1-G93A*) mice substantially accentuated ER stress and the associated pathology, rendering a previously resistant motor neuron subtype now vulnerable to ER dysfunction and disease manifestation. In the same vein, SIL1 expression was reduced in a mutant TDP-43 (*TDP-43^A315T^*) fALS murine model and co-localized with mutant TDP-43 in stress granule-like structures [101]. AAV-mediated SIL1 overexpression prevented denervation, reduced cellular stress, and increased survival, providing significance for this finding. In fact, total SIL1 levels were also found to be decreased in the cortex and hippocampus of a murine model of Alzheimer’s disease (Tg2576) [106], whereas the surviving hippocampal neuronal population in Alzheimer’s disease autopsies displayed higher SIL1 levels [107]. Overall, these results suggest a cytoprotective role of SIL1 in these neurodegenerative diseases.

While the expression of SIL1 demonstrates low cancer specificity, a recent study found that *SIL1* was expressed at higher levels in ~66% of glioblastoma multiforme and lower-grade gliomas compared to normal tissue, and it was particularly high in grade IV gliomas compared to grades I-III [108]. In fact, high expression of this gene in both glioblastoma multiforme and brain lower-grade gliomas is significantly associated (*p* < 0.001) with poor patient survival [108,109]. In keeping with a possible pathogenic role for SIL1 in gliomas, siRNA-mediated SIL1 knockdown in the glioblastoma cell line U251 caused a significant inhibition of cell proliferation and triggered apoptosis, although the mechanism by which this occurred has not been determined and warrants further investigation.

## 5. Potential Strategies to Treat MSS

Currently, no treatment regimens exist to target the pathological mechanisms underlying MSS. Instead, this disorder is managed symptomatically by cataract extraction or hormone replacement therapy for primary gonadal failure [110]. The logistical challenges for the discovery of new treatment options for MSS are similar to those for any rare disease, but the large number of distinct MSS-associated *SIL1* mutations, coupled with the variety of tissues affected and an incomplete understanding of the pathomechanisms of all primary defects, all hinder the exploration of potential therapeutic strategies for MSS. The two murine models and the recently developed zebrafish model of MSS offer important preclinical models for testing approaches that have been used for other protein folding diseases [76,86,87,111].

### 5.1. Chemical Chaperones

4-Phenylbutyrate (PBA) and tauroursodeoxycholic acid (TUDCA) are hydrophobic chemical chaperones whose proposed mechanisms of action involve their ability to interact with exposed hydrophobic regions in misfolded or unfolded proteins. Both of these chemical entities are orally bioavailable, have reasonable blood–brain barrier permeability with a no-to-low toxicity profile [112,113], have been used successfully in preclinical models for protein folding diseases [114,115], and are currently being evaluated in clinical trials for ALS [112,113,116,117]. Although plasma cell differentiation and antibody production are not dependent on SIL1 [75], MSS-derived LBLs that are deficient for SIL1 were more prone to cell death than normal controls when treated with tunicamycin, an ER stressor [118]. Pretreatment of these patient-derived LBLs with the chemical chaperone TUDCA decreased mitochondrial depolarization and caspase activation, and as a result, significantly reduced apoptotic cell death when the cells were subsequently exposed to tunicamycin [118]. However, in two other studies, pretreatment of MSS patient-derived LBLs with PBA had no major effect in decreasing ER stress-induced apoptotic cell death, despite numerous reports highlighting its ability to mitigate ER stress [119,120]. It is noteworthy that TUDCA is a bile acid, which can also mediate its cytoprotective effects by reducing ROS formation, thereby preventing mitochondrial dysfunction, and inhibiting apoptosis via the intrinsic and extrinsic pathways [121]. Plasma cells are particularly prone to ROS production due to the extremely high number of disulfide bonds required to fold and assemble an antibody molecule and the fact that each disulfide bond formed leads to the generation of a molecular oxygen species [122]. Although PBA had no effect in rescuing ER stress-induced death in the SIL1-deficient LBLs, it is not clear that other tissues would not be protected by this chemical chaperone, a possibility that requires further testing.

### 5.2. Gene Therapy for MSS

Gene therapy is a promising avenue for targeting monogenic diseases, which is the case for at least the 60% of MSS cases that are associated with *SIL1* mutations. Gene therapy has the potential to correct underlying genetic defects, with the prospect of requiring only a single dose to confer lifelong improvement [123]. Owing to the rapid advancement of this field and the development of next-generation viral vectors, demonstrating a desirable safety profile, broad tissue tropism, and evidence of clinical efficacy for some diseases, there are currently over 700 active gene therapy clinical trials currently underway. Thus, it is possible that gene therapy for MSS could also be clinically effective. A spectrum of adenovirus-associated viral-vector-based gene therapies with varying routes of administration are currently being pursued, from preclinical development through Phase IV trials, with the goal of targeting a wide variety of disorders, ranging from eye diseases, neurological/neurodegenerative diseases, and hemophilia to muscular dystrophy and others [124,125,126,127,128,129,130]. Successful pre-clinical results in numerous cases indicate that the necessary tissue tropism can be achieved as required for treating the tissues most affected in MSS, such as the central and peripheral nervous system, skeletal muscles, and the lens epithelia. 

### 5.3. Modulation of UPR Signaling

The studies of SIL1-deficient mice have provided ample evidence of the activation of the ER stress response in multiple tissues. The UPR is activated by three upstream transducers, all of which have been the target of small-molecule screens. The PERK-CHOP branch of the UPR has been demonstrated to be activated in cerebellar Purkinje cells [76], whereas the IRE1/XBP-1 arm of this response was activated in skeletal muscles of SIL1-deficient mice prior to their degeneration [86,87]. Prophylactic treatment of *Sil1^wz^* mice with a PERK inhibitor before the onset of cerebellar atrophy significantly delayed the emergence of physiological and biochemical signs of cerebellar degeneration and partially modulated the myopathy [131], which was shown to have a significant neurogenic contribution in this mouse model. However, the critical importance of the PERK pathway to pancreatic homeostasis makes this approach less attractive. Two other inhibitors, Sephin1 and Raphin1, have been identified that act downstream of PERK signaling to delay eIF2α dephosphorylation and prolong decreased protein synthesis. They have demonstrated promise in treating several animal models of proteostasis diseases, including Charcot-Marie-Tooth 1B syndrome, ALS, and Huntington’s disease [132,133], and show low toxicity. These molecules should be tested in animal models of MSS. While small molecule inhibitors of IRE1 [134] and ATF6 [135] are also available, it is possible that these inhibitors might actually exacerbate the pathologies associated with MSS. 

## 6. Conclusions

In the two decades since nucleotide exchange factors for BiP were discovered, mutations in one of them—SIL1—were found to be responsible for a majority of MSS cases. Structural data have uncovered the mechanism of nucleotide exchange, and the generation of animal models has provided insights into MSS-associated pathologies. While treatments for this multisystem disease are still lacking, the continued development of new strategies for combating protein folding diseases holds promise for the future.

## Data Availability

The datasets analyzed in this study are publicly available and can be found here: [proteinatlas.org/ENSG00000120725-SIL1].

## Abbreviations

ER	Endoplasmic Reticulum
ERdjs	ER-resident DnaJ-like Proteins
NEF	Nucleotide Exchange Factors
MSS	Marinesco-Sjögren Syndrome
ERQC	ER Protein Quality Control
HSP	Heat Shock Protein
UPS	Ubiquitin Proteasomal System
ERAD	ER-associated Degradation
CNX	Calnexin
CRT	Calreticulin
Ig	Immunoglobulin
UPR	Unfolded Protein Response
ATP	Adenosine Triphosphate
NBD	Nucleotide-binding Domain
SBD	Substrate-binding Domain
SRP	Signal Recognition Particle
Sil1	Suppressor of *Ire1* and *Lhs1* deletion 1
BAP	BiP-associated Protein
ARM	Armadillo-like Repeats
PBMCs	Peripheral Blood Mononuclear Cells
AARS	Alanyl tRNA Synthetase
IR	Insulin Receptor
IGF1R	Insulin-like Growth Factor Receptor
MIGIRKO	Muscle-Specific Dual Knockout of IR And IGF1R
NMJ	Neuromuscular Junctions
LBLs	Lymphoblastoid Lines
i.p.GTT	Intraperitoneal Glucose Tolerance Test
i.p.ITT	Intraperitoneal Insulin Tolerance Test
ALS	Amyotrophic Lateral Sclerosis
PHGDH	Phosphoglycerate dehydrogenase
ATXN10	Ataxin-10
PBA	4-Phenylbutyrate
TUDCA	Tauroursodeoxycholic Acid
SCA10	Spinocerebellar Ataxia Type 10
RIPK1	Receptor-interacting Serine/Threonine-Protein Kinase 1
